# Shifting paradigms of nontuberculous mycobacteria in cystic fibrosis

**DOI:** 10.1186/1465-9921-15-41

**Published:** 2014-04-11

**Authors:** Tavs Qvist, Tania Pressler, Niels Høiby, Terese L Katzenstein

**Affiliations:** 1 Department of Infectious Diseases, Cystic Fibrosis Center Copenhagen, Copenhagen University Hospital Rigshospitalet, Copenhagen, Denmark; 2 Department of Clinical Microbiology, University Hospital Rigshospitalet, Copenhagen, Denmark

**Keywords:** NTM, Nontuberculous mycobacteria, Cystic fibrosis, MABSC, MAC, Mycobacterium, Abcessus, Avium, Massiliense, Environmental mycobacteria, MOTT, Biofilm, Transmission, Lung transplantation

## Abstract

Important paradigms of pulmonary disease with nontuberculous mycobacteria (NTM) are currently shifting based on an increasing attention within the field of cystic fibrosis (CF). These shifts are likely to benefit the management of all patients with pulmonary NTM, regardless of underlying pathology. Currently several key areas are being revised: The first outbreak of human NTM transmission has been proven and new evidence of biofilm growth in vivo has been demonstrated. A better understanding of the clinical impact of NTM infection has led to increased diagnostic vigilance and new recommendations for lung transplantation are under way. While recent changes have reinvigorated the interest in NTM disease, the challenge remains, whether such advances can be successfully translated into improved management and care.

## Background

Announced as the most important emerging threat to cystic fibrosis (CF) patients at the 2013 European Cystic Fibrosis Conference, the perception of nontuberculous mycobacteria (NTM) disease has once again undergone a notable shift. Previously thought of as a group of rather benign environmental bacteria associated with random colonization and only rarely with genuine infection of the airway [1], perceptions have now changed. *Mycobacterium abscessus* Complex (MABSC) and *Mycobacterium avium* Complex (MAC) are now recognized as insidious opportunists that can seriously affect morbidity and mortality in CF. Fundamental paradigms of NTM pulmonary disease concerning clinical impact, patient susceptibility, transmission, bacterial modes of growth and implications for lung transplantation are currently being revised. Such issues are not unique to CF and can be expected to affect NTM management in patients with other underlying pulmonary diseases. While great leaps have been made in understanding the scope and impact of NTM infections, advances in diagnostics and antibiotic management have been less impressive and still await the benefits of the heightened attention NTM disease is now receiving. Since 2007, the American Thoracic Society (ATS)’s and the Infectious Disease Society of America (IDSA)’s comprehensive statement on NTM disease has been the principle guide of management [2]. 2014 appears to be the year where the eagerly expected CF specific NTM guidelines will be published, hopefully building further consensus in a field void of solid empirical evidence and highlighting areas in urgent need of attention.

### Biofilm growth

New evidence that rapidly growing NTM can grow *in vivo* as biofilms on intravascular catheters [3] could be an indication that biofilm growth in pulmonary NTM disease might also be a concern. Emerging evidence that pulmonary *Mycobacterium tuberculosis* (TB) is a biofilm infection [4] and our group’s recent examination of explanted CF lungs from patients with MABSC [5], suggests that this might indeed be the case. MABSC has been shown to be capable of structured cord formation, a biofilm mode of growth, associated with the morphologically distinct rough growth pattern [6,7]. This pattern is assumed to be similar to that proven for TB [8], suggesting that biofilm formation, is an inherent part of NTM pathogenesis in pulmonary disease. While phenotype switching of MABSC from smooth to rough has been linked to increased virulence [9], data on the genetic determinants of cord formation remain sparse. Future strategies might include using transposons for insertional mutagenesis [10], experimental lung models and sequential sequencing [11]. Addressing whether *in vivo* biofilm growth takes place in the CF lung could prove pivotal in explaining the notorious unreliability of *in vitro* susceptibility testing. The discrepancy between *in vitro* antibiotic resistance and the clinical benefit of treatment suggests that more than just antibiotic resistance is involved and that antibiotic tolerance, inferred by a protective biofilm coat could be present, in parallel with what is observed in other chronic infections [12,13]. Demonstrating NTM biofilm growth in the human lung could also open up new treatment opportunities as biofilm disrupting targets could be systematically examined, something that has not previously been attempted for NTM.

### Diagnostic challenges and opportunities

Identifying patients with NTM and clinical deterioration is difficult, but important, as these are the once most likely to benefit from antimycobacterial treatment. While conventional culture and acid-fast microscopy remain the backbone of mycobacterial diagnostics, some changes are under way, which could affect both the sensitivity and specificity of NTM diagnostics. New recommendations on NTM diagnostics are likely to include shorter time from sampling to culture, increased focus on routine screening, centralization of testing to larger mycobacteriological reference laboratories and validated pretreatment of samples to avoid Gram negative bacterial or fungal overgrowth. Several of these principles are already recommended, but not universally implemented [2]. MABSC, also called *Mycobacterium abscessus sensu lato* comprises the closely related species *Mycobacterium asbcessus sensu stricto*, *Mycobacterium massiliense* and *Mycobacterium bolletii*. Differentiation of the MABSC subspecies is difficult [14], and most clinical laboratories report infection with any of the three merely as *M. abscessus*. Historical variability in NTM taxonomy has only added to the confusion of how to correctly classify members of MABSC and MAC. Advances in molecular diagnostics and whole genome sequencing in particular, promise a brighter future for speciation, which is important in light of new insights into differences in virulence between subspecies. Importantly the subspecies *M. massiliense* remains susceptible to macrolides even after prolonged exposure, resulting from an inactivating erm (41) deletion [15]. Finally, immunological assays could prove useful as a means of monitoring NTM disease progression in a fashion similar to assays used in *Pseudomonas aeruginosa* infections in CF [16,17]. Thus, assays utilizing MABSC antigens-to-patient serum IgG have been shown to correlate with clinical disease [18,19] and are currently being explored further for clinical use [20].

### Prevalence and clinical impact

Reported prevalence rates have varied over time and geographically and are summarized in Table 1.

**Table 1:** Studies reporting the prevalence of nontuberculous mycobacteria in cystic fibrosis populations

**Study**	**Study year**	**Location**	**Design**	**Included CF patients**	**NTM cases**	**NTM revalence**	**% MABSC**	**% MAC**
Boxerbaum B.	1980	OH, USA	Prospective	430	8	1.8	75*	0
Smith MJ et al. [21]	1984	UK	Prospective	223	4	1.8	25*	0
Hjelte L et al. [22]	1990	Sweden	Prospective	54	5	9.2	0	60
Kilby JM et al. [23]	1992	NC, USA	Prospective	87	17	19.5	29*	76
Aitken ML et al. [24]	1993	WA, USA	Prospective	64	8	12.5	0	88
Hjelt K et al. [25]	1994	Denmark	Prospective	185	7	3.8	71*	29
Sermet-Gaudelus I et al. [26]	1996-99	France	Prospective	296	29	9.8	52	21
Fauroux B et al. [27]	1997	France	Prospective	106	7	6.6	43*	0
Mussaffi H et al. [28]	1997-02	Israel	Retrospective	139	12	8.6	67§	25§
Esther CR Jr et al. [29]	2000-07	NC, USA	Registry	1,216	166	13.7	41	59
Pierre-Audigier C et al. [30]	2000	France	Prospective	385	31	8.1	42	23
Oliver A et al. [19]	2000	Spain	Prospective	37	6	16.2	50*	33
Radhakrishnan et al. [31]	2004	Canada	Prospective	98	6	6.1	33	67
Levy I et al. [32]	2001-03	Israel	Cross-sectional	186	42	22.6	31	14
Olivier KN et al. [33]	2002	USA	Prospective	986	128	13.0	20	72
Roux AL et al. [34]	2004	France	Prospective	1,582	104	6.6	48	22
Valenza et al. [35]	2006	Germany	Prospective	60	8	13.3	50	50
Chalermskulrat W et al. [36]	2006	OH, USA	Retrospective	132	26	19.7	46	50
Binder AM et al. [37]	2011	USA	Registry	5,403	191	3.5	36	64

* = *M. chelonae* reported, but is here included as MABSC due to historical changes in taxonomy. § = percentage based on 6 chronic NTM cases.

Generally there has been a tendency towards higher prevalence rates being reported over time with a median prevalence rate of 9% (IQR: 3 – 11%) in the 9 studies initiated before 2000 and 13% (IQR: 7 – 17%) for the 10 post millennium prevalence studies. The reasons for this possible rise in NTM prevalence remain unknown, but could include changes in surveillance strategies although many centers are adamant that is not the case. Other reasons could include greater exposure [38], changes in the lung flora due to inhaled antibiotics [39], and reduced host immunity through autophagy inhibition during azithromycin treatment [40] or patient-to-patient transmission [15].

North American studies have consistently reported a higher proportion of MAC compared to MABSC [41], whereas the opposite is true for Western European studies [42], but the reasons for these geographical differences remain elusive. Another trend with some consistency, is that MABSC species, while isolated at all ages, seem to peak in prevalence in children, while MAC reaches the highest prevalence among adults [30,43,44], although this paradigm has been challenged [41]. Catherinot *et al*. have proposed that patient susceptibility might play a bigger role than previously thought, specifically pointing to an association between MABSC and more severe forms of CF [43]. Improved epidemiological surveillance such as the recent inclusion of NTM microbiology in the European CF registry will certainly be helpful in designing studies with the size required to address these questions.

While chronic MAC is not considered a critical threat to patient health, isolation of MABSC is often a real cause for concern. Some patients are diagnosed early on a routine mycobacterial culture, suggesting an initial phase of low to no clinical impact, which predates later loss of lung function [45]. Others develop chronic MABSC infection which can cause severe, sometimes fatal lung disease, even after an indolent period of several years [46]. A 2010 study from the US found that those infected with MABSC had a more rapid decline in lung function than their uninfected counterparts (3% per year vs. 2% per year) [29]. While the association with poor lung function is well established, the question of whether infection is mostly a consequence of, or a predisposing factor for poor lung function, remains unanswered. A 2014 study suggests that the rate of decline in FEV1 in the year leading up to the first positive culture may help distinguish patients who progress to active NTM disease and require treatment [41]. This study also reported that a quarter of patients cleared their NTM spontaneously [41] suggesting three phases of infection: Transient, persistent and active. Why some patients clear NTM suddenly without treatment, while others deteriorate rapidly warrants further investigation, as identifying who and when to treat remains the clinician’s central challenge in NTM management.

### Transmission

The first solid evidence of patient-to-patient transmission of NTM in a UK CF center was published in 2013 [15]. The authors used whole-genome sequencing and showed, with a reasonable degree of certainty that the MABSC subsp. *massiliense* had spread within their center. Out of 31 patients nine shared one genetically near-identical strain and two patients shared another strain. A convincing case for in- and out-patient overlap as well as careful elimination of potential sources of contamination (such as the water supply and bronchoscopy equipment) was performed. The study thus confirms suspicions raised in a report from Seattle, USA, that indicated possible transmission of a single strain of *M. massiliense*, identified through the use of pulse-field gel electrophoresis and PCR [47] and a similar, but less cited report from Sweden [48]. Mapping the genetic population structure of MABSC is an urgent issue currently being examined by Floto *et al*. in a multicenter study. This is expected to have significant impact on how infection control is managed in the in- and out-patient clinic.

### Lung transplantation

The view that NTM constitute an absolute contraindication for lung transplantation is being modified [49,50]. Reports on outcome following lung transplantation in CF patients with NTM infection are few and the impact on mortality remains unknown, although increased morbidity is consistently reported [49,51]. While aggressive treatment is recommended [2], eradication prior to transplantation is often unrealistic. Despite this, NTM disease does not preclude successful recovery after transplantation and new recommendations are expected to reflect this, bringing hope to a small, but growing number of patients, in desperate need of lung transplantation.

### Antibiotic treatment

Since the important shift in the 1990s away from antituberculous regimes towards macrolide based multidrug therapy [52], not much new has been accomplished in the field of antibiotic treatment of NTM. A part from a current phase 2 trial of inhaled amikacin in the US, a recent Cochrane Review concluded, that not a single randomized trial comparing antibiotic treatment of NTM lung infection in CF has been completed [53]. NTM are notoriously difficult to treat and require multidrug treatment for 12 months or more. At present, ATS guidelines are widely accepted as the standard of care [2], but there are several ongoing controversies about MAC and MABSC disease that could benefit from an updated consensus. The superiority of one macrolide over another has not been demonstrated for MAC lung disease, nor has the benefit of routinely including an IV drug (amikacin) early on been demonstrated [2] and both issues remain undecided for newly diagnosed MAC. A preliminary trial from a non-CF setting suggests that a two-drug regimen of clarithromycin and ethambutol may be non-inferior to the recommended three-drug regimen, which today includes rifampicin [54].

The duration and intensity of antibiotic treatment for MABSC, especially in patients that do not clear the bacteria, remains a headache for most clinicians. Inhaled therapy is increasingly used in MABSC regimens and a recent retrospective study of inhaled amikacin found that while reductions in smear positivity and some symptomatic improvement were observed, toxicity was not uncommon [55]. Hopefully, preliminary results from the ongoing phase 2 trial of inhaled liposomal amikacin will be released in 2014. New CF specific treatment guidelines under way are not expected to change the fundamental structure of past MABSC recommendations, which consist of an initial induction phase followed by long maintenance therapy, but new regimens will likely include longer induction phases and generally more antibiotics, with azithromycin as the macrolide of choice. NTM specific drug-drug interactions, medication side-effects and nonadherence are also areas receiving fresh attention.

## Conclusions

NTM seem to have found a successful niche in the structurally complex and often antibiotic rich environment of the CF lung. The central paradigms of how pulmonary NTM infection is initiated and how the disease progresses and interacts with the host have all shifted within the last years and upcoming consensus documents are eagerly awaited. While recent changes in the understanding of NTM disease have reinvigorated the field, the challenge remains, if such advances can be successfully translated into improved management and care.

## Abbreviations

CF: Cystic fibrosis; NTM: Nontuberculous mycobacteria; MABSC: *Mycobacterium abscessus* complex; MAC: *Mycobacterium avium* complex; ATS: American Thoracic Society; IDSA: Infectious Disease Society of America; TB: Tuberculosis; IV: Intravenous.

## Competing interests

The authors declare that they have no competing interests.

## Authors’ contributions

Manuscript was drafted by TQ and TLK. Concept was developed in collaboration with TP and NH who assisted in revision of the manuscript. All authors have seen and approved the finished manuscript. The manuscript has not previously been published nor is it being considered for publication elsewhere.
